# Dysfunction of the Murine Liver with Aging and Its Improvement with the Continuous Consumption of *Enterococcus faecalis* EC-12

**DOI:** 10.3390/nu16132031

**Published:** 2024-06-26

**Authors:** Yuko Makioka-Itaya, Ryo Inoue, Takamitsu Tsukahara

**Affiliations:** 1 Life Science Division, Combi Corporation, Saitama 338-0832, Japan; 2 Laboratory of Animal Science, Department of Applied Biological Sciences, Setsunan University, Hirakata 573-0101, Japan; ryo.inoue@setsunan.ac.jp; 3 Kyoto Institute of Nutrition & Pathology, Kyoto 610-0231, Japan; tsukahara@kyoto-inp.co.jp

**Keywords:** aging, chronic inflammation, *Enterococcus faecalis* EC-12, liver

## Abstract

Chronic inflammation is involved in the development of age-related diseases. Given its persistence, controlling chronic inflammation is essential for preventing age-related diseases. In this study, we investigated the effects of *Enterococcus faecalis* EC-12 (EC-12), which has immunomodulatory and antioxidant effects, on liver gene expression and aging phenomena in mice. Short-term EC-12 administration stimulated the expression of genes involved in lipid synthesis and metabolism in the liver. Furthermore, long-term EC-12 administration from 10 weeks to 1.5 years of age resulted in significant increases in blood interleukin (IL)-6 and IL-10 concentrations (both *p* < 0.05) and a significant decrease in the monocyte chemotactic protein-1 concentration (*p* < 0.05). These results indicated pathologic improvement, such as suppression of fat degeneration in the liver. These results suggest that continuous EC-12 intake from a young age can suppress liver function abnormalities, which is one of the aging phenomena in old age, and contribute to health in old age.

## 1. Introduction

Chronic inflammation is cited as a hallmark of human aging, and the activation of inflammasomes associated with chronic inflammation contributes to the development of aging-related diseases such as Alzheimer’s disease, atherosclerosis, macular degeneration, and osteoarthritis [1]. Inflammation is a biodefense mechanism that protects organisms from external stresses such as viral and bacterial infections, and it rapidly terminates after eliminating foreign bodies. However, chronic inflammation is characterized by a long-lasting and prolonged low-level inflammatory response in the absence of infection [2]. The cytokines secreted in chronic and acute inflammation differ. In particular, interleukin (IL)-2, IL-3, IL-4, IL-5, IL-7, IL-9, IL-10, IL-12, IL-13, IL-14, IL-15, interferons (IFNs), transforming growth factor (TGF)-β, and tumor necrosis factor (TNF)-β are characteristic of chronic inflammation. Meanwhile, IL-1, IL-6, IL-11, IL-17, TNF-α, eotaxin, and granulocyte-macrophage colony-stimulating factors are shared between acute and chronic inflammation. Controlling these cytokines is critical to suppressing chronic inflammation [3]. Oxidative stress control is also an essential component in preventing diseases associated with aging because reactive oxygen species (ROS) are also produced in inflammatory immune responses [4].

Lactic acid bacteria (LAB) and bifidobacteria, which live in abundance in the human intestine, have immunomodulatory effects, and they are expected to be effective against chronic inflammation associated with aging [5]. Reports are available on attempts to test LAB in elderly people to suppress chronic inflammation through its immunomodulatory effects. A 12-week study of enteral feeding of fermented milk containing *Lactobacillus johnsonii* La1 (NCC533) in older adults aged 75–96 years revealed a trend toward decreased blood TNF-α levels and a decrease in fever during the feeding phase, and Fukushima et al. suggested that these effects were attributable to the anti-inflammatory effect of *L. johnsonii* La1 (NCC533) [6]. Hyperhomocysteinemia is a cardiovascular risk factor attributable to chronic inflammation, such as that caused by myocardial infarction and ischemic cerebrovascular disorder. However, in a study in which nutritional guidance was given to healthy elderly people aged 65–85 years via the Internet, blood homocysteine levels were decreased after the completion of the study compared to those before the study in the group that received nutritional guidance and viable bacteria, whereas this phenomenon was not replicated in the group that received nutritional guidance alone [7].

Further in-depth analyses using animal models are in progress. In a study in which aged mice challenged with a high-fat diet were treated with a probiotic cocktail composed of five *Lactobacillus* and five *Enterococcus* strains, peritoneal macrophages collected from mice treated with the probiotic cocktail exhibited suppressed gene expression of proinflammatory cytokines, such as IL-6, TNF-α, and IL-1β, even after stimulation with lipopolysaccharide (LPS), compared to the findings in the control group, and similar reductions in gene expression were detected in colorectal tissue. In addition, increased gene expression of anti-inflammatory cytokines, such as IL-10 and TGF-β, was observed in the colon tissues of mice treated with the probiotic cocktail [8].

The chronic anti-inflammation effect of LAB has been reported for both live and inactivated bacteria. Heat-killed *Lactobacillus plantarum* OLL2712 improved hyperlipidemia by suppressing inflammatory cytokine levels in KK-Ay mice [9]. Heat-killed *L. plantarum* L-137 suppressed inflammatory mediators and biomarkers of lipid metabolism in healthy adult volunteers with an increased body mass index [10].

Although LAB consumption inhibits chronic inflammation by modulating the intestinal immune system, the liver, in particular, is likely to benefit from this strategy. In addition to being the central organ of metabolism in organisms, the liver is a site in which many immune cells accumulate [11]. Hepatic inflammation is associated with gut-derived toxic factors, as >70% of blood is delivered to the liver from the intestine through the portal vein [12]. For example, LPS derived from gut microbiota leaks from the gastrointestinal tract to the whole body through disruption of gut homeostasis, and LPS-induced TNF-α damages the liver and induces the development of nonalcoholic steatohepatitis [12], in which IL-17 levels in the intestine are increased, and liver inflammation is promoted by the migration of T-helper (Th) 17 cells from the intestine to the liver and the secretion of IL-17 [13]. Chronic inflammation in the liver can be sufficiently suppressed by maintaining intestinal immunity under normal conditions through LAB ingestion.

However, many attempts to suppress chronic inflammation via LAB consumption involved their ameliorative effects on subjects and animals with chronic inflammation, including elderly individuals [6,7], aged animals [8], obese individuals [10], and high-fat diet-fed mice [8]. To the best of our knowledge, there are no reports verifying the effects of LAB ingestion on the aging process before chronic inflammation develops.

We previously reported the various physiological effects of the heat-killed bacteria *Enterococcus faecalis* EC-12 (EC-12). In addition to immunomodulatory effects mediated by small intestinal Peyer’s patches [14] and IL-10 induction in antigen-presenting cells [15], we recently revealed that oral EC-12 consumption induces antioxidant effects (increased superoxide dismutase (SOD) activity) in the liver [16]. The effects of EC-12 ingestion over a long period from the juvenile stage to death have not been verified, although it can be expected to improve systemic chronic inflammation through the gut–liver axis via immunomodulatory effects involving intestinal immunity and SOD activity enhancement in the liver. Therefore, in this study, we comprehensively analyzed the changes in gene expression in the liver induced by EC-12 ingestion and verified the effectiveness of continuous EC-12 consumption on chronic inflammatory events that occur with aging. First, young mice were orally fed EC-12 (1 mg/kg/day) for 4 weeks, and the effects on hepatic gene expression were comprehensively verified. As the results revealed that hepatic gene expression was altered by EC-12 consumption, mice were then orally fed EC-12 (1 mg/kg/day) over a long period of time. At 1.5 years of age, the systemic inflammatory status was examined by the pathological evaluation of liver tissue and the measurement of cytokines in serum.

## 2. Materials and Methods

### 2.1. Experiment 1

Seven-week-old male BALB/c mice (SLC Japan, Shizuoka, Japan) were randomly divided into control and EC-12 groups based on their body weight (*n* = 16). The animals were housed in groups (*n* = 8) in plastic cages (345 × 403 × 177 mm^3^, CLEA Japan Inc., Tokyo, Japan) lined with wood chips under a 12 h/12 h light/dark cycle and a constant temperature of 25 °C. The cages were cleaned once a week. Acclimatized rearing was performed for 1 week. Breeding was performed by the group, and the feed during acclimatization was the AIN93M 20% casein diet (Oriental Yeast Co., Ltd., Tokyo, Japan). Feed and water were provided ad libitum throughout the study.

After acclimatization feeding, the control group continued to receive the AIN93M 20% casein diet, and the EC-12 group received the AIN93M 20% casein diet supplemented with 0.00125% EC-12 (the added concentration corresponds to EC-12 1 mg/kg/day) for 4 weeks. Heat-killed EC-12 (5.0 × 10^12^ cells/g) was manufactured by Combi Corporation (Saitama, Japan). Pentobarbital sodium (Somnopentyl; Kyoritsu, Tokyo, Japan) was used to anesthetize the mice after the completion of test diet feeding, and the livers of the animals were collected after sacrifice. The livers were stored at −80 °C after overnight immersion in RNA (Sigma-Aldrich Japan, Tokyo, Japan) until use.

Total RNA was extracted from the cryopreserved livers and subjected to DNA microarray analysis. The methods for extracting RNA and DNA microarrays were described by Inoue et al. (2018) [17]. MetaCore (Clarivate, Tokyo, Japan) was used for pathway analysis.

### 2.2. Experiment 2

Ten-week-old female C57BL6 mice (SLC Japan) were randomly divided into control and EC-12 groups based on their body weight (*n* = 16). The animals were housed in groups (*n* = 8) in plastic cages. The mice were kept in the same environment as that described in Experiment 1. Acclimatization was not performed, and the mice were reared in groups.

The control group was fed the AIN93M 20% casein diet, and the EC-12 group received the AIN93M 20% casein diet supplemented with 0.00125% EC-12 (EC-12 1 mg/kg/day) from the time of introduction until 1.5 years of age. Feed and drinking water were provided ad libitum throughout the study.

After the completion of the test diet feeding, pentobarbital sodium was used to anesthetize the mice, and their livers and abdominal vena cava blood were collected after sacrifice. A portion of the livers were immersed in a 10% neutral buffered formalin solution, and another portion was processed in the same manner as in Experiment 1 for DNA microarray. The serum was prepared from blood and stored at −80 °C until use.

After paraffin embedding and sectioning, organs were fixed in a 10% neutral buffered formalin solution, and hematoxylin and eosin (HE)-stained specimens were prepared. The generated HE-stained specimens were observed by light microscopy (BX-51; Olympus, Tokyo, Japan), and histopathology was performed. The detailed methods of HE staining were reported by Tsukahara et al. (2023) [16].

Inflammatory markers (IL-6, IL-10, monocyte chemotactic protein (MCP)-1, IFN-γ, TNF, and IL-12p70) in sera were measured using a Cytometric Bead Array (Mouse Inflammation Kit, BD, Tokyo, Japan).

### 2.3. Statistical Analysis

The outcome measure used for determining the sample size was JMP16.1.0 (JMP Japan, Tokyo, Japan). Assuming a 0.05 Type I error and a 0.10 Type II error (β = 90%), the number of mice required to obtain 90% certainty was six. Considering the uncertainties in animal experiments, the sample size was increased to eight animals per group.

Significant differences in the DNA microarray analysis were assessed, as described by Inoue et al. (2018) [17]. Additional statistical analyses were performed using the Mann–Whitney *U*-test and JMP16.1.0 (JMP Japan, Tokyo, Japan).

## 3. Results

### 3.1. Experiment 1

DNA microarray analysis of the liver revealed significant changes in expression for 91 genes in the EC-12 group compared to the control group, including 55 upregulated and 36 downregulated genes (Table S1, Figure S1a). Enrichment analysis was performed according to the results of gene expression analysis, and the pathways activated and suppressed by EC-12 feeding were identified (*p* < 0.05, Threshold 1, Table 1).

The pathways activated by EC-12 feeding included those related to fatty acid/cholesterol synthesis in the liver, such as “SREBP cleavage-activating protein (SCAP)/sterol regulatory element binding protein (SREBP) transcriptional control of cholesterol and fatty acid (FA) biosynthesis and cholesterol biosynthesis”, those related to lipid metabolisms, such as “regulation of lipid metabolism, regulation of acetyl-CoA carboxylase 1 activity, regulation of lipid metabolism via liver X receptor (LXR), nuclear factor-Y (NF-Y), and SREBP”, and “regulation of metabolism—bile acid regulation of glucose and lipid metabolism via farnesoid X receptor (FXR)”, and those related to oxidative stress, such as “glutathione metabolism” (Table 1). Pathways suppressed by EC-12 feeding compared to the control findings included pathways associated with “immune responses, such as IL-11 signaling via Janus tyrosine kinase (JAK)/signal transducer and activator of transcription (STAT)” and “immune response—IL-6-induced acute-phase response in hepatocytes” (Table 1).

### 3.2. Experiment 2

Of the 16 animals, one in the control group and two in the EC-12 group died before reaching 1.5 years of age; therefore, dissection at 1.5 years of age was performed on seven animals in the control group and six animals in the EC-12 group.

**Figure 1:**
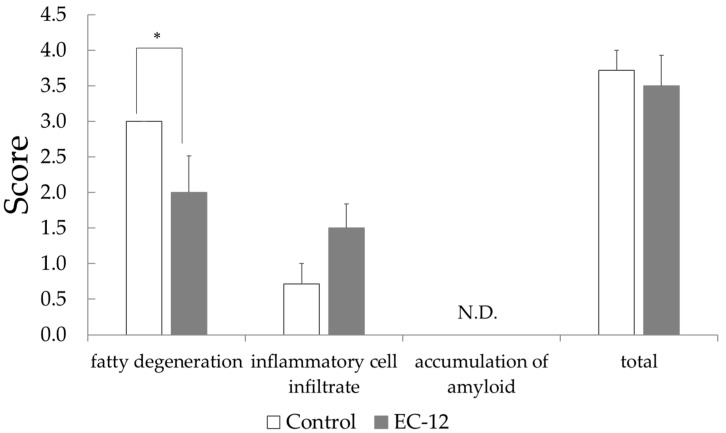
Pathological examination score in the liver of aged mice (Mean ± SD, *: *p* < 0.05).

Histopathological examination of the liver illustrated that the fatty degeneration score was 3.0 ± 0.0 in the control group versus 2.0 ± 0.5 in the EC-12 group (*p* < 0.05, Figure 1). In the control group, all mice had a steatosis score of 3, whereas in the EC-12 group, mice had a steatosis score of 2 to 0 (Figure S2). Conversely, the hepatic inflammatory cell infiltration score was 0.7 ± 0.3 in the control group, compared to 1.5 ± 0.3 in the EC-12 group, which was not significantly different (Figure 1). Liver amyloid deposition was not observed in any individual (Figure 1).

**Figure 2:**
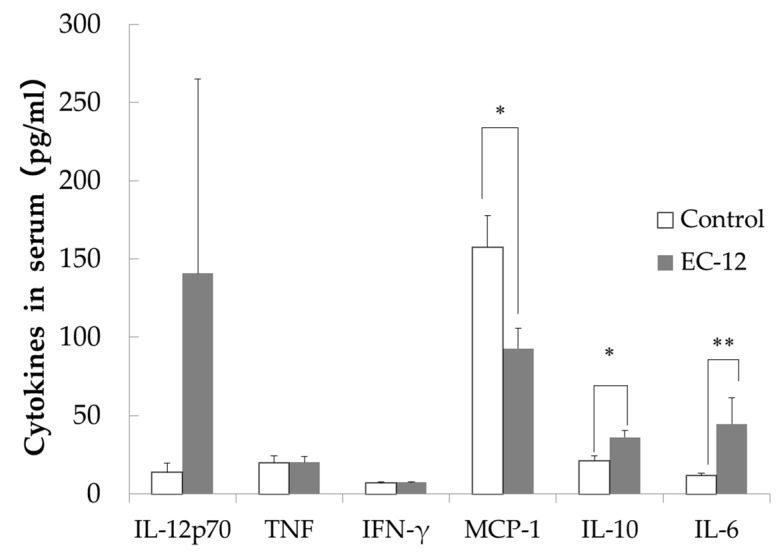
Inflammatory marker concentration in the serum of aged mice (Mean ± SD, *: *p* < 0.05, **: *p* < 0.01).

IL-12p70 levels in the sera were lower in the control group (13.7 ± 5.3 pg/mL) than in the EC-12 group (140.7 ± 124.3 pg/mL), although this difference did not reach significance (Figure 2). No differences in serum TNF or IFN-γ levels were observed between the groups (Figure 2). Serum MCP-1 levels were significantly higher in the control group (157.6 ± 19.9 pg/mL) than in the EC-12 group (92.8 ± 12.7 pg/mL, *p* < 0.05, Figure 2). In addition, serum IL-10 levels were significantly higher in the EC-12 group (36.1 ± 4.3 pg/mL) than in the control group (21.1 ± 3.1 pg/mL, *p* < 0.05, Figure 2). Serum IL-6 levels were significantly lower in the control group (11.6 ± 1.7 pg/mL) than in the EC-12 group (44.6 ± 16.8 pg/mL, *p* < 0.01, Figure 2).

A DNA microarray analysis of the liver revealed considerable changes in the expression of 56 genes in the EC-12 group compared with the control group, including 50 upregulated and 6 downregulated genes (Table S2, Figure S1b). Enrichment analysis was performed according to the results of the gene expression analysis, and the pathways that were activated and suppressed by EC-12 feeding were identified (*p* < 0.05, Threshold 1, Table S3). The pathways that were activated by EC-12 feeding included the pathway associated with “dysregulation of the germinal center response in systemic lupus erythematosus (SLE)” (Table S3). The pathways that were suppressed by EC-12 feeding compared with the control findings included the pathway associated with “G protein-coupled receptor signaling in lung cancer” (Table S3).

## 4. Discussion

When EC-12 was fed to young mice for 4 weeks (Experiment 1), pathways related to lipid synthesis in the liver, such as “SCAP/SREBP transcriptional control of cholesterol, FA biosynthesis, and cholesterol biosynthesis”, were activated. Meanwhile, metabolic pathways for lipids, such as “regulation of acetyl-CoA carboxylase 1 activity, regulation of lipid metabolism via LXR, NF-Y, and SREBP, and bile acid regulation of glucose and lipid metabolism via FXR”, were also activated (Table 1). In addition, “glutathione metabolism” was activated, and the pathway of active oxygen elimination was enhanced. Conversely, pathways related to liver inflammation, such as “IL-11 signaling via JAK/STAT and IL-6-induced acute-phase responses in hepatocytes”, were suppressed. Several reports describe the activation of host lipid metabolism following probiotic administration. For example, oral administration of *Lactobacillus sakei* MJM60958 was reported to reduce the gene expression of acetyl-CoA carboxylase and SREBP-1 in the livers of high-fat diet-fed mice [18]. FXR, a receptor for bile acids, is expressed on cells associated with innate immunity, such as hepatic macrophages (Kupffer cells), and FXR activation inhibits inflammation by the Nod-like receptor (NLR) family pyrin domain containing 3 [19]. As previously described, EC-12 has immunomodulatory effects on the intestinal tract and spleen, suggesting that EC-12 feeding activates hepatic immune cells through the gut–liver axis to stimulate FXR expression and regulate the activity of the hepatic adipose synthetic system. Glutathione metabolism reduces oxidative stress, and aging is accelerated by glutathione deficiency [20]. The increased glutathione metabolism activity in the livers of young mice following EC-12 administration for 4 weeks might have contributed to a reduction in hepatic oxidative stress. The oxidative stress-reducing effect of EC-12 is consistent with previous findings [16]. ROS and reactive nitrogen species (RNS) are produced by cellular inflammatory responses. Excessive ROS and RNS production leads to lipid oxidation, resulting in the development of metabolic syndrome [21]. In high-fat diet-induced obesity, hepatic inflammation resulting from sustained IL-6 or IL-11 activation leads to steatohepatitis and hepatitis [22,23]. Although the diet of the mice used in Experiment 1 was normal but not a high-fat diet, EC-12 administration suppressed hepatic inflammation induced by IL-6 and IL-11, and SOD activity in the liver was increased by EC-12, resulting in reduced oxidative stress and suppressed lipid oxidation in the liver. In a small study of human subjects, EC-12 consumption reduced body fat accumulation in men with metabolic syndrome [24]. 

It has been confirmed that short-term *Lactococcus lactis* subsp. *lactis* JCM 5805 activates plasmacytoid dendritic cells (pDC) [25], whereas long-term administration improves the viability of senescence-accelerated mouse prone 6 (SAMP6) mice, a senescence-accelerated strain [26], and reduces the senescence score by continuously activating pDC. As we could confirm in Experiment 1, EC-12 intake caused changes in liver gene expression, and long-term continuous intake of EC-12 also affected the damage that accumulates in the liver with aging. Based on this hypothesis, we conducted Experiment 2. In mice with long-term EC-12 consumption, we observed reductions in hepatic steatosis and serum MCP-1 levels and increases in serum IL-6 and IL-10 levels compared to the findings in the control group (Experiment 2). Fatty degeneration of the liver is caused by the accumulation of aged hepatocytes triggered by decreased mitochondrial function [27]. As fatty degeneration of the liver progresses with age, metabolic-dysfunction-associated steatotic liver disease (MASLD) develops. MASLD was previously termed nonalcoholic fatty liver disease (NAFLD); however, in 2023, NAFLD was renamed MASLD at the initiative of the American, European, Asian Pacific, and Latin American Association for the Study of the Liver [28]. Hereafter, when studies from before 2023 are cited, they are referred to as NAFLD, as they were at the time of publication. In animal experiments, LAB has been reported to have a preventive effect against high-fat diet-induced NAFLD, and its anti-inflammatory effect has been suggested as the underlying mechanism. The symptoms of patients with NAFLD improved after the ingestion of LAB [29]. Chronic administration of d-galactose to animals causes conditions similar to natural aging, such as the accumulation of reactive acid species and glycation, whereas *L. plantarum* DR7 and *Lactobacillus fermentum* DR9 suppress fat accumulation and fatty degeneration in the liver of d-galactose-induced aging rats fed a high-fat diet [30]. Although mice were not fed a high-fat diet in this study, the age-related accumulation of fat in the liver was confirmed (Figure 1). Therefore, the inhibition of fatty degeneration in the mouse liver by long-term feeding of EC-12 contributed to the potential prevention of MASLD. It has been confirmed that intestinal mucosal MCP-1 levels increased with age in SAMP8 mice, and oral administration of *Lactobacillus paracasei* PS23 for 12 weeks prevented the increase in intestinal mucosal MCP-1 levels in mice [31]. In this study, EC-12 administration appeared to suppress aging-related increases in MCP-1 levels, and EC-12 had similar suppressive effects on inflammation as *L. paracasei* PS23, attributable to its effects on blood MCP-1 levels (Figure 2). The significant increases in serum IL-6 and IL-10 levels with long-term EC-12 feeding warrant discussion. First, systemic IL-10 production increases with aging. This is attributable to the feedback regulation of chronic inflammatory conditions associated with aging [32]. IL-10 production in response to microbial stimuli also appears to be higher in aged mice, and it has been reported that IL-10 production is more strongly induced by LPS administration in mice at approximately 100 weeks of age than in mice at approximately 7 weeks of age [33]. This suggests that the long-term feeding of EC-12 can enhance systemic IL-10 production in aged mice. In a report revealing that *L. paracasei* PS23 administration in SAMP8 mice blocked the reduction of cognitive function with aging, serum IL-10 levels were also increased by *L. paracasei* PS23 administration, and the cerebral function of SAMP8 mice was maintained by suppressing aging-association inflammation through the anti-inflammatory effect of IL-10 [34]. Increased serum IL-10 levels upon long-term EC-12 feeding can be considered to enhance anti-inflammatory effects, as reported by Huang et al. (2018) [34], in conjunction with the inhibition of hepatic steatosis in aged mice. This is because IL-10 is also associated with hepatic inflammation and adiposity. High-fat diet tolerance tests in splenectomized mice with IL-10 KO revealed that IL-10 inhibits hepatic adiposity [35]. EC-12 has been demonstrated to induce IL-10 production in human peripheral blood mononuclear cells [15]. These results suggest that increased serum IL-10 levels attributable to long-term EC-12 consumption contributed to the inhibition of hepatic steatosis associated with aging. Similarly, IL-6 has been reported to be associated with aging as a marker of chronic inflammatory conditions, such as cognitive decline [36] and physical performance decline [37], and IL-6 has also been reported to contribute to improved hepatic glucose tolerance. A few reports state that IL-6 improves glucose tolerance in obese mice by acting on the central nervous system and suppressing feeding behavior [38]. In addition, insulin resistance and glucose intolerance are observed in mice with liver-specific IL-6Rα KO, resulting in increased IL-6, IL-10, and TNF-α expression. However, it has been reported that insulin resistance and glucose intolerance are improved by eliminating Kupffer cells or neutralizing TNF-α [39]. The current study revealed that hepatic IL-6 can suppress inflammatory cytokines such as TNF-α to exert anti-inflammatory effects and improve glucose tolerance. In addition, IL-6 contributed to the anti-inflammatory effect, suggesting that it suppressed age-related fatty degeneration of the liver, in line with previous findings that exercise-induced IL-6 might have an anti-inflammatory effect because it induces IL-10 [40].

Two discrepancies are observed between the results of Experiments 1 and 2. First, in Experiment 1, lipid synthesis and lipid metabolism were promoted in the liver, whereas in Experiment 2, only the suppression of fatty degeneration was observed. The enhanced synthesis of lipids in the liver also reflects an event other than lipid accumulation in this organ. Liu et al. (2014) reported that fatty acids are degraded in skeletal muscles during the daytime, whereas they are synthesized in the liver at night [41]. This suggests that fat, which is synthetically activated by EC-12 administration, is efficiently used for energy production in skeletal muscles, thereby promoting metabolism and consequently reducing fat accumulation in the aged liver, although this finding requires further examination in the future. Second, in Experiment 1, the administration of EC-12 suppressed the “immune response—IL-6-induced acute-phase response in hepatocytes” in the liver, whereas in Experiment 2, the serum IL-6 level was elevated by feeding EC-12. In Experiment 1, the enhancement of IL-6-related pathways was confirmed, whereas IL-6 gene expression in the liver and the serum IL-6 level were not. In Experiment 2, the effect of long-term EC-12 intake on gene expression in the liver was more modest than that recorded in Experiment 1, and it activated the pathway of “immune response—IL-6-induced acute-phase response in hepatocytes” (Tables S2 and S3, Figure S1b). Then, considering the explanations of the opposing results obtained in Experiments 1 and 2, the effects obtained from (1) EC-12 intake changed with life stage changes, and (2) different durations of EC-12 administration had different effects on the liver. In fact, in a study in which probiotic VSL#3 was administered to young and aged rats for 6 weeks, the effects of VSL#3 on brain gene expression differed between young (3-month-old) and aged (20–22-month-old) rats [42]. Immune function changes with life stage transition [43], suggesting that EC-12 exerts its effects on liver gene expression and systemic inflammation via host gut immunity, which indicates that the effects obtained from EC-12 feeding may also have changed with life stage transition. In an experiment in which milk fermented with *Lactobacillus* sp. and *Streptococcus* sp. was orally administered to mice for 98 days, the number of IgA-positive cells and cytokine-positive cells on days 2–98 were measured. The counts of the intestinal tract in the fermented milk group were considerably higher than those in the control group throughout the study period. Conversely, the IgA-positive cell counts in the fermented milk group of the bronchus-associated lymphoid tissue and the mammary gland were considerably higher in the early stage of treatment (1 week) than in the control group. However, the difference between the control and fermented milk groups disappeared thereafter, and no substantial differences in cytokine-positive cell counts were observed at any time point [44]. In that study, the phagocytic activity of peritoneal macrophages, which signal to sites away from the intestinal tract after long-term administration of fermented milk, was also evaluated. The phagocytic activity was considerably increased compared with the control group up to 2 weeks after the onset of fermented milk administration, after which the difference from the control group was resolved. This suggests that the liver was affected by gene expression via peritoneal macrophages during the short-term administration of EC-12 but was affected by EC-12 administration via a different mechanism.

## 5. Conclusions

The present study revealed that EC-12 altered the expression of genes involved in hepatic lipid synthesis, lipid metabolism, oxidative stress responses, and inflammation after 4 weeks of feeding, and protein- and tissue-level changes were observed after long-term EC-12 feeding into old age, resulting in the suppression of chronic inflammation and hepatic steatosis. This is the first study to demonstrate that EC-12 feeding enhances both hepatic lipid synthesis and lipid metabolism. The findings also indicated that the effect of EC-12 was induced via the control of chronic inflammation through its immunomodulatory effects. However, room exists for further research because (1) this was a small study and (2) only 1.5 years of age data were obtained for Experiment 2. Therefore, although changes over time cannot be mentioned, this study suggests that continuous intake of lactic acid bacteria, such as EC-12, from a young age suppresses chronic inflammation in old age and improves health in old age. The fact that the mechanism via which EC-12 affects the organism varies with the duration of the administration and age is a subject for future study.

## Figures and Tables

**Table 1:** Pathway analysis results of genes affected by EC-12 in the liver of young mice.

Maps	Total	*p*-Value	FDR	In Data	Network Objects from Active Data
Activated					
SCAP/SREBP transcriptional control of cholesterol and FA biosynthesis	45	4.469 × 10^−10^	3.754 × 10^−8^	6	ELOVL6, IDI1, INSIG1, GPAM, ACACA, LSS
Cholesterol biosynthesis	103	7.214 × 10^−8^	3.030 × 10^−6^	6	IDI1, NSDHL, CYP51A1, ER24, SC4MOL, LSS
Glutathione metabolism	71	1.568 × 10^−5^	4.390 × 10^−4^	4	GSTM1, GSTA5, GSTM5, G6PD
Regulation of lipid metabolism—regulation of acetyl-CoA carboxylase 1 activity	18	6.858 × 10^−4^	1.440 × 10^−2^	2	ACACB, ACACA
Insulin-dependent stimulation of SREBP-1 in type 2 diabetes in the liver	27	1.554 × 10^−3^	2.510 × 10^−2^	2	INSIG1, ACACA
Adiponectin in the pathogenesis of type 2 diabetes	29	1.793 × 10^−3^	2.510 × 10^−2^	2	ACACB, ACACA
Regulation of lipid metabolism—regulation of lipid metabolism via LXR, NF-Y, and SREBP	38	3.068 × 10^−3^	3.139 × 10^−2^	2	CYP51A1, ACACA
PXR-mediated direct regulation of xenobiotic metabolizing enzymes/rodent version	39	3.229 × 10^−3^	3.139 × 10^−2^	2	ELOVL6, S14 protein
Regulation of metabolism—bile acid regulation of glucose and lipid metabolism via FXR	41	3.564 × 10^−3^	3.139 × 10^−2^	2	ACACB, ACACA
PXR-mediated direct regulation of xenobiotic metabolizing enzymes/human version	42	3.737 × 10^−3^	3.139 × 10^−2^	2	ELOVL6, S14 protein
suppressed					
Immune response—IL-11 signaling via JAK/STAT	34	5.956 × 10^−6^	2.442 × 10^−4^	3	Metallothionein-I (rodent), Myeloblastin, Pim-3
Immune response—IL-6-induced acute-phase response in hepatocytes	36	6.781 × 10^−4^	9.267 × 10^−3^	2	SAA1, SAA3

In the table, pathways that were clearly not related to the liver were not considered, and only pathways in which the expression of two or more genes was observed are listed. SCAP: SREBP cleavage-activating protein; SREBP: sterol regulatory element binding protein; FA: fatty acid; LXR: liver X receptor; NF-Y: nuclear factor-Y; FXR: farnesoid X receptor; JAK/STAT: Janus tyrosine kinase/signal transducer and activator of transcription.

## Data Availability

The original contributions presented in the study are included in the article, further inquiries can be directed to the corresponding author.

## Supplementary Materials

The following supporting information can be downloaded at: https://www.mdpi.com/article/10.3390/nu16132031/s1, Table S1. Gene expression affected by EC-12 in the liver of young mice. Table S2. Gene expression affected by EC-12 in the liver of aged mice. Table S3. Pathway analysis results of genes affected by EC-12 in the liver of aged mice. Figure S1. Alteration of the gene-expression profile caused by EC-12 administration in the liver of (a) young mice (*n* = 8 in each group) and (b) aged mice (*n* = 7: in the control group; *n* = 6: in the EC-12 group). Figure S2. Microscopic images of fatty degeneration in the liver of aged mice (H&E staining, magnification ×40, bar 20 μm).
