# Supplementary material for: Dysfunction of the Murine Liver with Aging and Its Improvement with the Continuous Consumption of Enterococcus faecalis EC-12
Source: Nutrients. 2024 Jun 26;16(13):2031. doi: 10.3390/nu16132031 (PMC11243158; doi:10.3390/nu16132031)
Supplement: Supplementary file 1 [file nutrients-16-02031-s001.zip › nutrients-3047824-supplementary.pdf]

Table S1. Gene expression affected by EC-12 in the liver of young mice.

Feature ID	Annotations - Gene symbol	Annotations - Description	Control	EC-12	EC12 -Control
**Activated by EC-12 treatment**					
A_52_P350750	*Chrna4*	Mus musculus cholinergic receptor, nicotinic, alpha polypeptide 4 (Chrna4), mRNA [NM_015730]	3.87	6.57	2.70
A_55_P2018666	*Thrsp*	Mus musculus thyroid hormone responsive (Thrsp), mRNA [NM_009381]	12.08	14.18	2.10
A_51_P463440	*Elovl6*	Mus musculus ELOVL family member 6, elongation of long chain fatty acids (yeast) (Elovl6), mRNA [NM_130450]	11.38	13.32	1.94
A_55_P2078494	*Cib3*	Mus musculus calcium and integrin binding family member 3 (Cib3), mRNA [NM_001080812]	5.97	7.74	1.77
A_52_P164161	*Cyp51*	Mus musculus cytochrome P450, family 51 (Cyp51), mRNA [NM_020010]	7.37	9.06	1.69
A_55_P2025514	*Pnpla3*	Mus musculus patatin-like phospholipase domain containing 3 (Pnpla3), mRNA [NM_054088]	6.10	7.76	1.66
A_55_P2046411		predicted gene 7979 [Source:MGI Symbol;Acc:MGI:3648893] [ENSMUST00000223009.1]	13.18	14.83	1.65
A_55_P2026761		predicted gene 5873 [Source:MGI Symbol;Acc:MGI:3648385] [ENSMUST00000177157.1]	12.71	14.34	1.63
A_66_P137462	*Nsdhl*	Mus musculus NAD(P) dependent steroid dehydrogenase-like (Nsdhl), mRNA [NM_010941]	8.41	10.00	1.59
A_51_P187082	*G6pdx*	Mus musculus glucose-6-phosphate dehydrogenase X-linked (G6pdx), mRNA [NM_008062]	8.97	10.52	1.56
A_55_P2028961	*Idi1*	Mus musculus isopentenyl-diphosphate delta isomerase (Idi1), mRNA [NM_145360]	11.52	13.06	1.54
A_55_P2168267		Unknown	13.12	14.62	1.50
A_30_P01023533		lincRNA:chr8:126268510-126290810 reverse strand	6.77	8.25	1.48
A_51_P209372	*Msmo1*	Mus musculus methylsterol monoxygenase 1 (Msmo1), mRNA [NM_025436]	11.53	13.00	1.47
A_55_P2019058	*Acaca*	Mus musculus acetyl-Coenzyme A carboxylase alpha (Acaca), mRNA [NM_133360]	12.70	14.12	1.42
A_51_P290207	*Insig1*	Mus musculus insulin induced gene 1 (Insig1), mRNA [NM_153526]	8.31	9.72	1.42
A_55_P2099840		predicted gene 10642 [Source:MGI Symbol;Acc:MGI:3704338] [ENSMUST00000098589.2]	6.88	8.28	1.40
A_30_P01026769		lincRNA:chr3:45081952-45092352 forward strand	5.79	7.18	1.39
A_55_P2161456	*Serpina3a*	Mus musculus serine (or cysteine) peptidase inhibitor, clade A, member 3A (Serpina3a), transcript variant 1, mRNA [NM_001167705]	6.73	8.12	1.39
A_55_P2041723	*Mid1ip1*	Mus musculus Mid1 interacting protein 1 (gastrulation specific G12-like (zebrafish)) (Mid1ip1), transcript variant 1, mRNA [NM_001166635	11.36	12.68	1.32
A_30_P01025049		lincRNA:chr3:93582250-93592950 reverse strand	4.79	6.04	1.25
A_55_P2078955	*Aqp8*	Mus musculus aquaporin 8 (Aqp8), transcript variant 1, mRNA [NM_007474]	10.35	11.60	1.25
A_55_P1976395	*Nsdhl*	Mus musculus NAD(P) dependent steroid dehydrogenase-like (Nsdhl), mRNA [NM_010941]	6.42	7.66	1.24
A_55_P2001474	*Klk1b26*	Mus musculus kallikrein 1-related petidase b26 (Klk1b26), mRNA [NM_010644]	5.61	6.85	1.24
A_55_P2002933	*Klk1b5*	Mus musculus kallikrein 1-related peptidase b5 (Klk1b5), mRNA [NM_008456]	7.38	8.61	1.24
A_55_P2293917	*1700007F19Rik*	Mus musculus RIKEN cDNA 1700007F19 gene (1700007F19Rik), long non-coding RNA [NR_040538]	4.66	5.89	1.23
A_51_P296487	*Lss*	Mus musculus lanosterol synthase (Lss), mRNA [NM_146006]	8.05	9.24	1.19
A_52_P120122		predicted gene 20661 [Source:MGI Symbol;Acc:MGI:5313108] [ENSMUST00000176382.7]	2.91	4.10	1.19
A_52_P539161	*Rdh11*	Mus musculus retinol dehydrogenase 11 (Rdh11), transcript variant 1, mRNA [NM_021557]	10.61	11.80	1.19
A_55_P2044453		Unknown	5.01	6.19	1.18
A_55_P2025006	*Egfbp2*	Mus musculus epidermal growth factor binding protein type B (Egfbp2), mRNA [NM_010115]	7.14	8.31	1.17
A_51_P456208	*Tff3*	Mus musculus trefoil factor 3, intestinal (Tff3), mRNA [NM_011575]	5.47	6.63	1.16
A_55_P2019054	*Acacb*	Mus musculus acetyl-Coenzyme A carboxylase beta (Acacb), mRNA [NM_133904]	12.07	13.23	1.16
A_55_P2122841		Unknown	14.06	15.21	1.16
A_55_P2171406	*Tm7sf2*	Mus musculus transmembrane 7 superfamily member 2 (Tm7sf2), mRNA [NM_028454]	11.00	12.16	1.15
A_30_P01024808		lincRNA:chr8:126268510-126290810 reverse strand	5.11	6.26	1.15
A_55_P2121392	*Acaca*	Mus musculus acetyl-Coenzyme A carboxylase alpha (Acaca), mRNA [NM_133360]	6.75	7.87	1.12
A_51_P245414	*Klk1*	Mus musculus kallikrein 1 (Klk1), transcript variant 1, mRNA [NM_010639]	6.69	7.80	1.11
A_55_P2318584	*Aqp8*	Mus musculus aquaporin 8 (Aqp8), transcript variant 1, mRNA [NM_007474]	14.65	15.75	1.10
A_55_P2156963	*Cyp11b1*	Mus musculus cytochrome P450, family 11, subfamily b, polypeptide 1 (Cyp11b1), mRNA [NM_001033229]	3.20	4.29	1.09
A_55_P2085400	*Gpam*	Mus musculus glycerol-3-phosphate acyltransferase, mitochondrial (Gpam), transcript variant 2, mRNA [NM_001356285]	8.49	9.58	1.09
A_55_P2134004	*Gstm2*	Mus musculus glutathione S-transferase, mu 2 (Gstm2), mRNA [NM_008183]	11.97	13.05	1.08
A_55_P2016039		Unknown	3.94	5.01	1.07
A_55_P2170454	*Gsta2*	Mus musculus glutathione S-transferase, alpha 2 (Yc2) (Gsta2), mRNA [NM_008182]	8.41	9.47	1.06
A_55_P2112315	*6330444E15Rik*	PREDICTED: Mus musculus RIKEN cDNA 4930432E11 gene (4930432E11Rik), misc_RNA [XR_035433]	2.02	3.08	1.06
A_55_P2031671	*Gstm6*	Mus musculus glutathione S-transferase, mu 6 (Gstm6), mRNA [NM_008184]	11.56	12.61	1.05
A_66_P130035	*Klk1b24*	Mus musculus kallikrein 1-related peptidase b24 (Klk1b24), mRNA [NM_010643]	7.34	8.39	1.05
A_55_P1953387	*Fabp5*	Mus musculus fatty acid binding protein 5, epidermal (Fabp5), transcript variant 2, mRNA [NM_001272097]	14.86	15.91	1.05
A_30_P01028766		lincRNA:chr9:78107225-78118850 forward strand	8.61	9.65	1.04
A_66_P124420		predicted gene 7049 [Source:MGI Symbol;Acc:MGI:3646400] [ENSMUST00000223520.1]	9.19	10.23	1.04
A_52_P384574	*Stard4*	Mus musculus StAR-related lipid transfer (START) domain containing 4 (Stard4), transcript variant 1, mRNA [NM_133774]	10.68	11.72	1.04
A_52_P627068	*Disp2*	Mus musculus dispatched RND tramsporter family member 2 (Disp2), mRNA [NM_170593]	3.15	4.18	1.03
A_55_P2111855	*Gale*	Mus musculus galactose-4-epimerase, UDP (Gale), mRNA [NM_001356493]	11.16	12.18	1.02
A_55_P2305420	*D9Wsu90e*	AY673530 NADH dehydrogenase subunit 4 {Malacochersus tornieri} (exp=-1; wgp=0; cg=0), partial (9%) [TC1621524]	9.41	10.42	1.01
A_55_P2084703	*Acaca*	Mus musculus acetyl-Coenzyme A carboxylase alpha (Acaca), mRNA [NM_133360]	5.43	6.44	1.00
**Suppressed by EC-12 treatment**					
A_51_P371750	*Marco*	Mus musculus macrophage receptor with collagenous structure (Marco), mRNA [NM_010766]	8.93	7.92	-1.01
A_55_P2202357	*0710001A04Rik*	Q5XJM0_BRARE (Q5XJM0) Zgc:101785, partial (5%) [TC1706550]	5.59	4.57	-1.02
A_55_P2063256	*Lgals4*	Mus musculus lectin, galactose binding, soluble 4 (Lgals4), mRNA [NM_010706]	11.16	10.14	-1.02
A_55_P2050044		Unknown	13.28	12.25	-1.03
A_30_P01019750		lincRNA:chr17:32044823-32045974 reverse strand	10.09	9.04	-1.05
A_30_P01030993		lincRNA:chr6:31017987-31174287 forward strand	4.22	3.16	-1.06
A_55_P2028986	*Lbp*	lipopolysaccharide binding protein [Source:MGI Symbol;Acc:MGI:1098776] [ENSMUST00000109491.7]	8.96	7.89	-1.07
A_30_P01024995		lincRNA:chr17:32044823-32045974 reverse strand	9.60	8.48	-1.11
A_55_P2282361	*Ctcflos*	Mus musculus CCCTC-binding factor (zinc finger protein)-like, opposite strand (Ctcflos), long non-coding RNA [NR_040321]	8.04	6.90	-1.14
A_30_P01030988		lincRNA:chr16:90103524-90104408 reverse strand	8.74	7.58	-1.16
A_55_P2183433	*Rab30*	Mus musculus RAB30, member RAS oncogene family (Rab30), mRNA [NM_029494]	8.70	7.54	-1.17
A_30_P01021437		lincRNA:chr17:32035750-32056000 reverse strand	10.10	8.93	-1.17
A_55_P1994112	*Sema5b*	Mus musculus sema domain, seven thrombospondin repeats (type 1 and type 1-like), transmembrane domain (TM) and short cytoplasmic d	6.24	5.04	-1.19
A_66_P106760	*Adam32*	Mus musculus a disintegrin and metallopeptidase domain 32 (Adam32), transcript variant 1, mRNA [NM_153397]	7.09	5.88	-1.21
A_51_P189746	*Pim3*	Mus musculus proviral integration site 3 (Pim3), mRNA [NM_145478]	10.32	9.10	-1.22
A_55_P2037033	*Rgs22*	Mus musculus regulator of G-protein signalling 22 (Rgs22), mRNA [NM_001195748]	3.57	2.30	-1.27
A_55_P2039429	*Dio1*	Mus musculus deiodinase, iodothyronine, type I (Dio1), mRNA [NM_007860]	6.96	5.61	-1.35
A_52_P318673	*Saa1*	Mus musculus serum amyloid A 1 (Saa1), transcript variant 1, mRNA [NM_009117]	16.84	15.39	-1.46
A_55_P2004447	*Fgl1*	Mus musculus fibrinogen-like protein 1 (Fgl1), mRNA [NM_145594]	15.21	13.73	-1.48
A_51_P209818	*Prtn3*	Mus musculus proteinase 3 (Prtn3), mRNA [NM_011178]	7.17	5.67	-1.50
A_55_P1970033	*Per1*	Mus musculus period circadian clock 1 (Per1), transcript variant 1, mRNA [NM_011065]	9.93	8.40	-1.53
A_55_P1983921	*Crybb3*	Mus musculus crystallin, beta B3 (Crybb3), transcript variant 3, mRNA [NM_001359004]	8.87	7.28	-1.59
A_55_P2187918	*Cep55*	Mus musculus centrosomal protein 55 (Cep55), transcript variant 2, mRNA [NM_028760]	7.71	6.06	-1.65
A_52_P294510	*Fgl1*	Unknown	14.92	13.27	-1.65
A_51_P279437	*Mfsd2a*	Mus musculus major facilitator superfamily domain containing 2A (Mfsd2a), mRNA [NM_029662]	10.95	9.30	-1.65
A_55_P2105140	*Mrap2*	Mus musculus melanocortin 2 receptor accessory protein 2 (Mrap2), transcript variant 3, mRNA [NM_001359955]	6.17	4.49	-1.69
A_55_P1953169	*Saa3*	Mus musculus serum amyloid A 3 (Saa3), mRNA [NM_011315]	10.81	8.95	-1.86
A_55_P2295762	*4933433G15Rik*	Unknown	2.19	0.29	-1.90
A_55_P2046657	*Dio1*	Mus musculus deiodinase, iodothyronine, type I (Dio1), mRNA [NM_007860]	8.45	6.45	-2.01
A_51_P269792	*Rad51b*	Mus musculus RAD51 paralog B (Rad51b), transcript variant 1, mRNA [NM_009014]	8.99	6.79	-2.21
A_51_P424532	*Vnn1*	Mus musculus vanin 1 (Vnn1), mRNA [NM_011704]	9.93	7.64	-2.29
A_52_P423810	*Mt1*	metallothionein 1 [Source:MGI Symbol;Acc:MGI:97171] [ENSMUST00000211807.1]	8.57	6.13	-2.44
A_66_P111660	*Mt1*	Mus musculus metallothionein 1 (Mt1), mRNA [NM_013602]	15.99	13.46	-2.53
A_51_P311958	*Orm3*	Mus musculus orosomucoid 3 (Orm3), mRNA [NM_013623]	10.85	8.28	-2.57
A_55_P2070869	*Lcn2*	Mus musculus lipocalin 2 (Lcn2), mRNA [NM_008491]	13.28	10.65	-2.63
A_55_P1999902	*Pip5k1a*	phosphatidylinositol-4-phosphate 5-kinase, type 1 alpha [Source:MGI Symbol;Acc:MGI:107929] [ENSMUST00000107229.1]	13.87	10.93	-2.94

Table S1 shows 91 genes whose gene expression was changed by feeding EC-12 in the livers of young mice. In the livers of young mice in the EC-12 group, the expression of 55 genes was increased and the expression of 36 genes was decreased compared to the control group. Genes that showed differences due to feeding EC-12 were defined as genes with an absolute difference in gene expression between the groups of 1 or more.

Table S2. Gene expression affected by EC-12 in the liver of aged mice.

Feature ID	Annotations - Gene symbol	Annotations - Description	Control	EC-12	EC12 -Control
**activated by EC-12 treatment**					
A_51_P461067		immunoglobulin heavy constant gamma 1 (G1m marker) [Source:MGI Symbol;Acc:MGI:96446] [ENSMUST00000103420.2	5.29	7.91	2.62
A_55_P2060897		immunoglobulin kappa constant [Source:MGI Symbol;Acc:MGI:96495] [ENSMUST00000103410.2]	12.01	14.55	2.54
A_55_P1977451		immunoglobulin kappa variable 4-80 [Source:MGI Symbol;Acc:MGI:4439653] [ENSMUST00000103341.3]	6.17	8.68	2.51
A_55_P2058023		immunoglobulin kappa variable 4-57-1 [Source:MGI Symbol;Acc:MGI:2686264] [ENSMUST00000103356.3]	6.47	8.97	2.50
A_52_P429450	*Ngp*	Mus musculus neutrophilic granule protein (Ngp), mRNA [NM_008694]	4.45	6.84	2.39
A_66_P140474		immunoglobulin kappa chain variable 4-70 [Source:MGI Symbol;Acc:MGI:2686348] [ENSMUST00000103348.2]	6.85	9.23	2.38
A_55_P2186978		immunoglobulin kappa variable 4-62 [Source:MGI Symbol;Acc:MGI:3643587] [ENSMUST00000198345.1]	6.60	8.98	2.38
A_51_P395899		immunoglobulin kappa variable 3-1 [Source:MGI Symbol;Acc:MGI:1330851] [ENSMUST00000103404.1]	6.26	8.55	2.28
A_55_P2186972		immunoglobulin kappa variable 4-79 [Source:MGI Symbol;Acc:MGI:2685040] [ENSMUST00000103342.3]	5.45	7.72	2.27
A_52_P15388	*Ltf*	Mus musculus lactotransferrin (Ltf), mRNA [NM_008522]	5.79	8.00	2.21
A_55_P2003911		immunoglobulin kappa variable 6-17 [Source:MGI Symbol;Acc:MGI:1330833] [ENSMUST00000103391.3]	6.25	8.39	2.14
A_30_P01024145		lincRNA:chr17:29378878-29379589 forward strand	6.99	9.07	2.07
A_55_P2011950		immunoglobulin heavy variable 1-85 [Source:MGI Symbol;Acc:MGI:3645723] [ENSMUST00000103552.1]	5.09	7.04	1.96
A_55_P2103948		immunoglobulin kappa variable 4-53 [Source:MGI Symbol;Acc:MGI:2686266] [ENSMUST00000198328.1]	5.99	7.89	1.90
A_66_P136569		immunoglobulin heavy variable 1-39 [Source:MGI Symbol;Acc:MGI:4439888] [ENSMUST00000103515.1]	4.27	6.17	1.90
A_55_P1993001		immunoglobulin heavy variable 1-55 [Source:MGI Symbol;Acc:MGI:4439716] [ENSMUST00000103526.2]	5.30	7.18	1.88
A_55_P2001269		immunoglobulin heavy variable 1-61 [Source:MGI Symbol;Acc:MGI:4439824] [ENSMUST00000103531.3]	5.65	7.51	1.86
A_55_P2023717	*Kcnq5*	potassium voltage-gated channel, subfamily Q, member 5 [Source:MGI Symbol;Acc:MGI:1924937] [ENSMUST0000011529	1.62	3.45	1.83
A_66_P113466		immunoglobulin kappa chain variable 4-90 [Source:MGI Symbol;Acc:MGI:4439830] [ENSMUST00000103334.3]	5.68	7.51	1.82
A_55_P2001267		Unknown	3.84	5.66	1.82
A_55_P2186942		immunoglobulin kappa variable 10-94 [Source:MGI Symbol;Acc:MGI:3646140] [ENSMUST00000103330.1]	3.89	5.69	1.79
A_66_P106497		immunoglobulin kappa chain variable 8-30 [Source:MGI Symbol;Acc:MGI:3642250] [ENSMUST00000103378.2]	5.15	6.84	1.69
A_55_P2001274		immunoglobulin heavy variable 1-62-3 [Source:MGI Symbol;Acc:MGI:3648544] [ENSMUST00000103532.2]	5.65	7.34	1.69
A_66_P109692		immunoglobulin kappa variable 4-92 [Source:MGI Symbol;Acc:MGI:2686254] [ENSMUST00000103332.1]	4.66	6.32	1.66
A_55_P2138627		immunoglobulin heavy variable 6-3 [Source:MGI Symbol;Acc:MGI:4439854] [ENSMUST00000103486.1]	3.35	5.00	1.65
A_51_P288295		immunoglobulin heavy variable V1-7 [Source:MGI Symbol;Acc:MGI:3704122] [ENSMUST00000103496.3]	6.09	7.72	1.63
A_51_P232281	*Pla2g2d*	Mus musculus phospholipase A2, group IID (Pla2g2d), transcript variant 1, mRNA [NM_011109]	4.61	6.10	1.49
A_30_P01024923		lincRNA:chr18:47327300-47333819 forward strand	2.61	4.03	1.42
A_55_P2057283		immunoglobulin lambda constant 2 [Source:MGI Symbol;Acc:MGI:99547] [ENSMUST00000103749.2]	9.28	10.69	1.42
A_55_P2153517	*Enho*	Mus musculus energy homeostasis associated (Enho), mRNA [NM_027147]	11.17	12.57	1.40
A_51_P104768		immunoglobulin heavy variable V1-9 [Source:MGI Symbol;Acc:MGI:4439621] [ENSMUST00000193893.5]	5.22	6.61	1.39
A_55_P2068461	*Htr2c*	Mus musculus 5-hydroxytryptamine (serotonin) receptor 2C (Htr2c), mRNA [NM_008312]	2.16	3.49	1.33
A_55_P2187030		immunoglobulin heavy variable 1-66 [Source:MGI Symbol;Acc:MGI:4439825] [ENSMUST00000103537.2]	5.32	6.62	1.30
A_66_P128763	*LOC102636989*	predicted gene 498 [Source:MGI Symbol;Acc:MGI:2685344] [ENSMUST00000207642.1]	2.38	3.67	1.29
A_55_P2140348		immunoglobulin heavy variable 1-55 [Source:MGI Symbol;Acc:MGI:4439716] [ENSMUST00000103526.2]	4.09	5.37	1.28
A_55_P2018254		predicted gene 10295 [Source:MGI Symbol;Acc:MGI:3642770] [ENSMUST00000094315.2]	2.36	3.62	1.26
A_51_P342652	*Cd79b*	Mus musculus CD79B antigen (Cd79b), transcript variant 1, mRNA [NM_008339]	8.93	10.13	1.20
A_55_P1953169	*Saa3*	Mus musculus serum amyloid A 3 (Saa3), mRNA [NM_011315]	9.96	11.15	1.19
A_55_P2106121	*Il21*	Mus musculus interleukin 21 (Il21), transcript variant 1, mRNA [NM_001291041]	3.35	4.52	1.17
A_55_P1970464		immunoglobulin heavy variable 1-22 [Source:MGI Symbol;Acc:MGI:4439784] [ENSMUST00000103507.1]	5.11	6.28	1.17
A_52_P538084		immunoglobulin kappa variable 1-135 [Source:MGI Symbol;Acc:MGI:3819952] [ENSMUST00000103303.2]	3.97	5.09	1.11
A_52_P184368	*Ncam2*	Mus musculus neural cell adhesion molecule 2 (Ncam2), transcript variant 2, mRNA [NM_010954]	2.25	3.35	1.10
A_52_P223654	*Rsg1*	REM2 and RAB-like small GTPase 1 [Source:MGI Symbol;Acc:MGI:1923416] [ENSMUST00000151475.1]	2.34	3.43	1.09
A_52_P1190151		immunoglobulin kappa variable 10-96 [Source:MGI Symbol;Acc:MGI:4439561] [ENSMUST00000103328.2]	4.25	5.32	1.07
A_55_P2026258		immunoglobulin kappa variable 12-46 [Source:MGI Symbol;Acc:MGI:4439773] [ENSMUST00000103365.2]	6.05	7.11	1.07
A_55_P2123491	*B3gnt5*	Mus musculus UDP-GlcNAc:betaGal beta-1,3-N-acetylglucosaminyltransferase 5 (B3gnt5), transcript variant 1, mRNA [NM_	4.24	5.30	1.06
A_55_P1988994		immunoglobulin kappa chain variable 1-122 [Source:MGI Symbol;Acc:MGI:4439722] [ENSMUST00000103314.2]	5.26	6.31	1.05
A_55_P2090330	*Kcnmb4*	Mus musculus potassium large conductance calcium-activated channel, subfamily M, beta member 4 (Kcnmb4), mRNA [NM	3.34	4.37	1.03
A_55_P1964648	*Btla*	Mus musculus B and T lymphocyte associated (Btla), transcript variant 1, mRNA [NM_001037719]	6.56	7.58	1.03
A_55_P1968953	*Mycs*	Mus musculus myc-like oncogene, s-myc protein (Mycs), mRNA [NM_010850]	2.61	3.64	1.03
**suppressed by EC-12 treatment**					
A_51_P187625	*Cabp2*	Mus musculus calcium binding protein 2 (Cabp2), transcript variant 1, mRNA [NM_013878]	6.34	5.33	-1.01
A_55_P2050226	*Ackr4*	AY072938 chemokine receptor CCX CKR {Mus musculus} (exp=-1; wgp=0; cg=0), complete [TC1596586]	7.32	6.28	-1.04
A_55_P2113758	*Gria1*	Mus musculus glutamate receptor, ionotropic, AMPA1 (alpha 1) (Gria1), transcript variant 1, mRNA [NM_001113325]	4.50	3.41	-1.09
A_51_P458852	*Ina*	Mus musculus internexin neuronal intermediate filament protein, alpha (Ina), mRNA [NM_146100]	6.20	5.09	-1.11
A_55_P2138796	*A530016L24Rik*	Mus musculus RIKEN cDNA A530016L24 gene (A530016L24Rik), mRNA [NM_177039]	3.10	1.98	-1.11
A_51_P514405	*Slc2a5*	Mus musculus solute carrier family 2 (facilitated glucose transporter), member 5 (Slc2a5), mRNA [NM_019741]	9.44	8.11	-1.33

Table S1 shows 56 genes whose gene expression was changed by feeding EC-12 in the livers of aged mice. In the livers of aged mice in the EC-12 group, the expression of 50 genes was increased and the expression of 6 genes was decreased compared to the control group. Genes that showed differences due to feeding EC-12 were defined as genes with an absolute difference in gene expression between the groups of 1 or more.

Table S3. Pathway analysis results of genes affected by EC-12 in the liver of aged mice.

Maps	Total	p-value	FDR	In Data	Network Objects from Active Data
**Activated**					
Dysregulation of germinal center response in SLE	65	1.029E-03	5.044E-02	2	CD79B, IL-21
**Suppressed**					
G protein-coupled receptors signaling in lung cancer	76	3.966E-04	4.760E-03	2	Galpha(q)-specific peptide GPCRs, Galpha(i)-specific peptide GPCRs

In the table, only pathways in which expression of two or more genes was observed are listed.

SLE : systemic lupus erythematosus

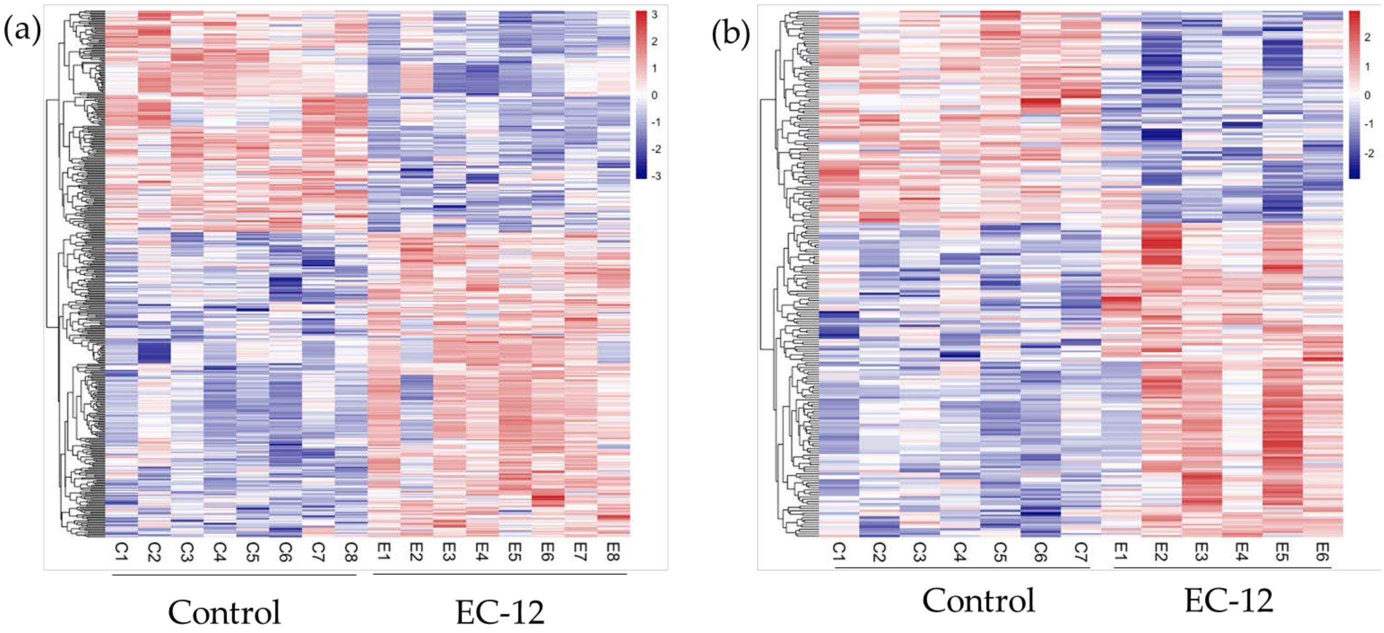

Figure S1. Alteration of the gene-expression profile caused by EC-12 administration in the liver of (a) young mice (n=8 in each group) and (b) aged mice (n = 7: in the control group; n=6 : in the EC-12 group).

The heat map shows the expression values (log2 transformed) of the genes that were significantly altered in the EC-12 group compared with the control group ($p < 0.05$, $-1 \leq$ fold change ≥ 1) .

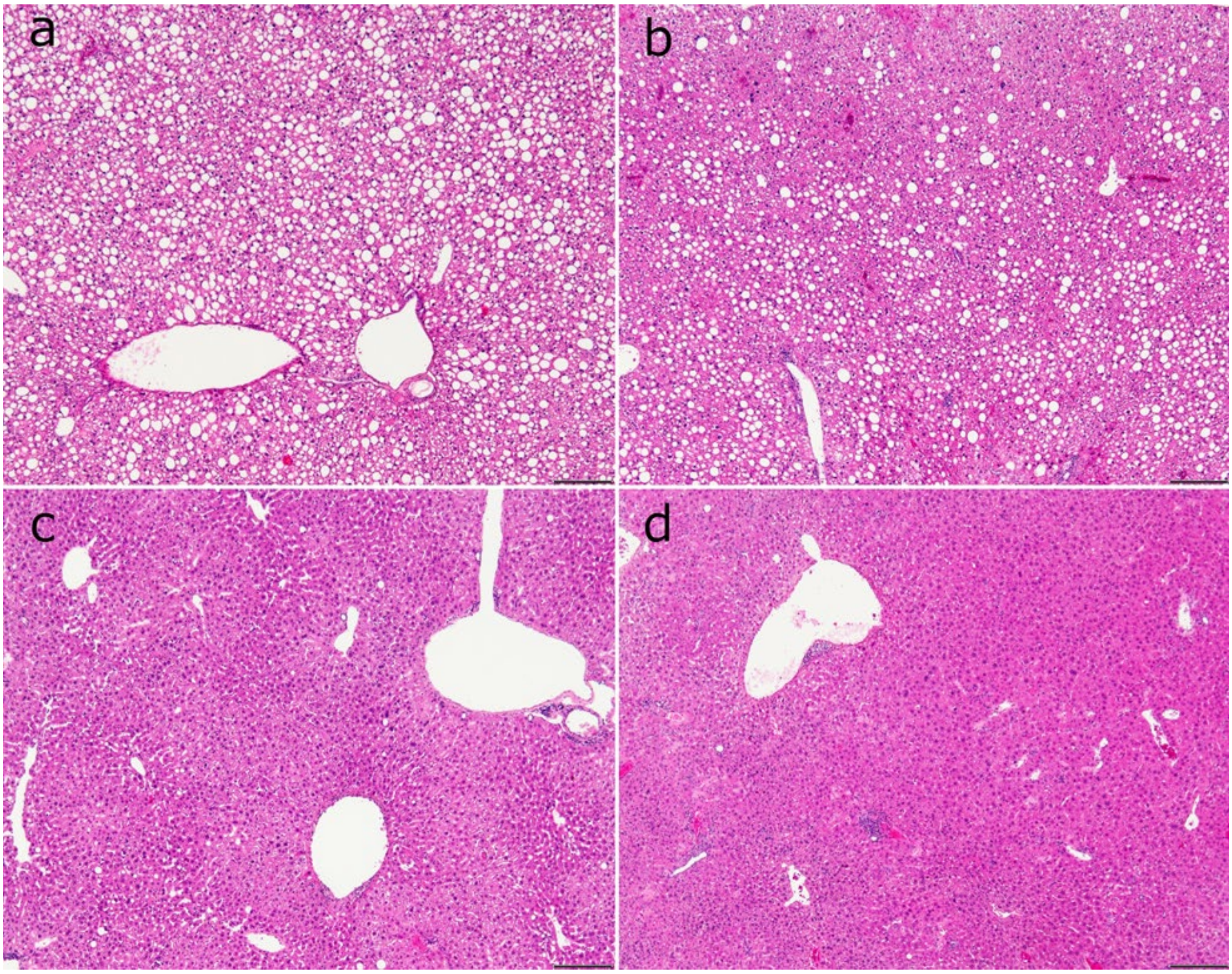

Figure S2. Microscopic images of fatty degeneration in the liver of aged mice (H&E staining, magnification ×40, bar 20 μm).

(**a**) Control (fatty degeneration score : 3) (**b**) EC-12 (fatty degeneration score : 2)

(**c**) EC-12 (fatty degeneration score : 1) (**d**) EC-12 (fatty degeneration score : 0)
